# Navigating the patient-provider relationship during severe illness with lessons from a qualitative perspective of calciphylaxis

**DOI:** 10.1038/s41598-025-09116-6

**Published:** 2025-07-02

**Authors:** Olivia G. Durant, Sagar U. Nigwekar, Carmel Salhi

**Affiliations:** 1 Kidney Research Center, Massachusetts General Hospital, Boston, MA USA; 2 Bouvé College of Health Sciences, Northeastern University, Boston, MA USA; 3 Division of Nephrology, Department of Medicine, Massachusetts General Hospital, Boston, MA USA

**Keywords:** Calciphylaxis, Pain, Qualitative perspective, Severe illness, Patient-provider relationship, Kidney diseases, Skin diseases, Nephrology, Patient education, Quality of life

## Abstract

Calciphylaxis is a condition with unpredictable onset that predominantly affects people with kidney disease. This rare, incredibly painful condition results in necrotic skin lesions caused by calcified occlusions of the microvasculature and has an average one-year mortality rate of 50%. There is no cure for calciphylaxis, and treatment primarily focuses on symptom management. We sought to understand the lived experience of calciphylaxis. This qualitative study utilized semi-structured, phenomenological interviewing. We created a code table in which primary codes were analyzed from the biopsychosocial perspective from which overarching domains of experience were identified. Our sample consisted of 15 outpatient participants: 9 were in remission from calciphylaxis while 6 had active disease. Twelve participants were women while 3 were male. Participants ages ranged from 38 – 80 (mean 59). The calciphylaxis patient experience was characterized by severe pain, a lack of adequate pain management, limited calciphylaxis literacy among providers, and complicated interpersonal relationships with family, friends, and medical providers. Calciphylaxis is a life-altering condition characterized by severe pain and uncertainty on multiple fronts. This study demonstrates that clinical management of rare, severe illnesses, such as calciphylaxis, requires clear, honest provider-patient communication, sufficient provider and patient education, adequate symptom management, and empathy.

## Introduction

Calciphylaxis is a rare condition with poorly understood pathogenesis that predominantly affects people with kidney disease and causes non-healing, necrotic skin ulcers due to calcified occlusions of the microvasculature in subcutaneous adipose and dermis tissue^1,2^. Healing of calciphylaxis wounds proves difficult, leading to excruciating pain and one-year mortality rate of 50% on average^1,3^. The incidence of calciphylaxis has been reported to be on the rise^1,4^. Treatment of calciphylaxis is resource intensive, multidisciplinary, and most effective when the condition is promptly recognized^3,5,6^.

Given that a cure for calciphylaxis does not exist and treatment mainly focuses on symptom management^1,7^, understanding how patients live with the condition proves important. This study, which aims to investigate the lived experience of calciphylaxis using phenomenological, semi-structured interviews, may provide useful information to providers. Our goal is to describe the lived experience of calciphylaxis in ways that will aid a clinician’s understanding of calciphylaxis patients themselves, which aids a patient-centered approach to care^8^. Eventually, we hope these results will impact calciphylaxis management and research by highlighting what is of importance to calciphylaxis patients. Through developing a deeper, more comprehensive understanding of what it is like to live with calciphylaxis, we also aim to provide information that may improve not only the lived experience of calciphylaxis but also other severe, acute conditions.

## Methods

### Study design

This qualitative study utilized semi-structured, phenomenological interviewing. Interviews were conducted between October 2022 and April 2023. The study was approved by the Mass General Brigham Institutional Review Board (IRB #2022P001182).

### Participant selection and recruitment

Using purposive sampling, we interviewed 15 outpatients with the diagnosis of calciphylaxis made within 10 years of the interview month. These patients were recruited through a registry of patients (ClinicalTrials.gov ID: NCT03032835) at a clinical practice in the Northeastern United States who had been seen by experts in managing calciphylaxis, who had agreed to be contacted for research, or who had contacted the provider requesting to participate in research. All human subjects research was conducted in accordance with institutional guidelines and regulations. Recruitment continued until thematic saturation, defined as the point when data analysis does not yield any new themes or add to the existing understanding of previously defined themes, in the domains of clinical experience, social support, and emotional response, was reached.

### Data collection

We utilized a phenomenological approach to developing an interview guide to allow participants to recall, in as much depth as possible, specific events and experiences^9^. An interview guide addressing three domains of experience, initial calciphylaxis diagnosis, nature of care, diagnosis, and treatment of calciphylaxis, and social/daily effects of calciphylaxis, was developed (Table 1). The guide contained open-ended questions regarding the calciphylaxis experience that allowed participants to focus on recalling and describing experiences^10^.

**Table 1:** Interview guide summary.

Domain of data collection	Example question topics
Initial diagnosis of calciphylaxis	- Initial symptoms - Initial clinical encounters
Nature of care, treatment, and management of calciphylaxis	- Different ways in which one received care - Providers involved in care
Social/Daily effects of calciphylaxis	- Coping - Social support

Participants were emailed an informed consent form and a brief interview guide before interviewing. Informed consent was obtained from each participant prior to beginning the interview. Demographic information was collected from the medical record or at the end of interviews. Author OD conducted all interviews using video conference (Zoom, Google Duo) or telephone. Author OD was trained in qualitative methods by author CS and is a nephrology research assistant to author SUN. Interviews ranged from 45 to 90 minutes in duration with breaks allowed. All interviews were audio recorded and verbatim transcribed by Landmark Associates, LLC.

### Data analysis

The goal of our analysis was to understand the lived experiences of people with calciphylaxis and develop a narrative around participants’ calciphylaxis experiences. To understand the reality of living with calciphylaxis, we utilized a biopsychosocial (BPS) perspective. The biopsychosocial model emphasizes disease’s biological, psychological, and social aspects. Using this model as a guide, our analysis focused on the pain experience, the experience of uncertainty related to calciphylaxis prognosis and treatment, and the experience of interacting with clinicians treating calciphylaxis. The biopsychosocial model emphasizes how a participant’s psychological factors impact their healing and recovery process and social functioning, which is appropriate to consider in the context of calciphylaxis due to the severe nature of the disease^11^.

Coding began after interview transcription. Utilizing a phenomenological approach guided by the BPS model, transcripts were reviewed line by line to develop open codes relating to participants’ experiences until saturation of experience was reached. Next, we collapsed the open codes into a thematic codebook (Table 2), which included primary code labels, definitions of codes, and exemplar quotations. Authors OD and CS independently open-coded 4 and 3 interviews, respectively, and compared open codes to discuss discrepancies and improve reliability before creating the thematic codebook. Open codes were also shared with author SUN, the study’s clinical expert, to verify clinical experience. We then used NVivo 12.7.0 for data management and further interview analysis. Each transcript was read, line by line, and coded by OD according to the primary code labels. The next step in our analysis was to create a code table in which primary codes were analyzed from the biopsychosocial perspective. From here, overarching domains of experience were identified.

**Table 2:** Thematic codebook summary.

Thematic domain	Brief definition	Codes
Uncertainty	*The unpredictability and limited biological and social understandings of calciphylaxis, including disease course, treatment course, and general knowledge, and resignation to the ambiguity of the condition*	- Clinician ambivalence - Disease course unpredictability - Personal experience of understanding calciphylaxis and disease prognosis uncertainty
Pain	*The experience and attempted management, and sequelae thereof, of physical pain related to calciphylaxis wounds and disease*	- Descriptions of pain - Addressing pain management - Impact of pain on function
Support	*The actions, roles, and emotional investment, or lack thereof, of others experienced during a person’s experience with calciphylaxis, from disease development to current state*	- Actors involved or absent - The ways in which support is or is not demonstrated - Reactions to support
Personal experience of disease	*The process of emotionally responding to having calciphylaxis*	- Coping - Emotional response - Making sense of one’s calciphylaxis (outside of coping): - Impacts of dialysis on calciphylaxis experience
Clinical care	*The process and varied manners in which patients receive clinical/medical care for their calciphylaxis, including the sharing of information regarding calciphylaxis, the communication of providers, the array of treatments, and attitudes towards calciphylaxis*	- Familiarity and knowledge - Approach to treatment and planning - Engagement in treatment process

## Results

### Descriptive statistics

Our sample consisted of 15 outpatient participants: 9 were in remission from calciphylaxis while 6 had active disease. Eight participants received their calciphylaxis diagnosis while receiving either hemodialysis or peritoneal dialysis. Two participants who were not on kidney replacement therapy were started on hemodialysis as part of calciphylaxis treatment. The remaining 5 participants did not receive dialysis. Three participants identified as male while the remaining 12 identified as female. All participants were fluent in English. See Table 3.

**Table 3:** Patient characteristics (n = 15).

Characteristic	Mean (range)
Age	59 (34–80)
**Gender**	**n**
Female	12
Male	3
**Race/Ethnicity**	**n**
African American/Black, Non-Hispanic	3
Other, Non-Hispanic	1
White, Hispanic	1
White, Non-Hispanic	10
**Dialysis use at time of calciphylaxis diagnosis**	**n**
Yes	8
No	7
**Calciphylaxis distribution**	**n**
Central* Only	1
Peripheral** Only	12
Central and peripheral	2

* = Calciphylaxis present on the truck of the body.

** = Calciphylaxis present on the extremities or limbs (arms or legs).

Note: Comprehensive information on all comorbidities and socioeconomic factors were not available.

### Pain as the motivation to seek initial medical care

Across all participants, pain was the symptom that motivated their initial desire to seek care. Participants described the pain as unsettling and significantly disruptive to their lives because of its severity, its disproportionality to lesion size, or its unknown origin. One participant noted *“a small wound on my left leg…It was very little, almost nothing. I remember it hurt a lot more than a wound that size should hurt”* (12). Another participant described how the unknown onset was unsettling: *“It was a pain where it hurt to move [my legs] as if maybe I worked out…but I hadn’t been working out. The pain freaked me out” (3).* The pain’s combination of severity, sudden onset, and unknown origin was a signal that something was awry with the participants’ bodies.

Though some participants attempted to self-treat their pain using commonly available analgesic medications, the pain often resulted in behavioral changes. As one participant described, “*the pain was so intense that I couldn’t walk normally… I’m wearing flare pants, things that won’t touch my skin because the pain is intense” (15).* The rapid worsening and severity of the pain led participants to seek medical attention: *“Finally, I was like, “I can’t do this pain anymore. Nothing is helping.” … We finally went to the ER, and I was admitted immediately” (1).* For all participants, pain was the most salient early symptom of calciphylaxis, which later posed challenges in its clinical management and disruption to their day-to-day lives. Though the pain was the driving factor to seek care, the generalized nature of the pain, along with varying severity and accompanying lesions, often led participants to different initial points of contact with the medical system.

### Initial point of contact and (Mis) diagnosis

Participants’ first point of contact for care was usually a general practitioner, a dermatologist, an emergency department clinician, or a nephrologist. Many participants received misdiagnoses, including but not limited to deep vein thrombosis, cellulitis, and shingles. Participants noted their skepticism with initial diagnoses as they did not align with the severity of their pain or the types of lesions they were experiencing, leading to dissatisfaction with the diagnosis or conclusions from their first medical encounters.

Participants often had to be proactive in correcting initial misdiagnoses. In an outpatient context, participants described a cycle of seeing a provider, trying a treatment, getting worse, and then going back to the same or a different doctor for a new treatment until they came across a provider who either recognized calciphylaxis or could refer the participant to the correct specialist. One participant described the process of seeking out specialist care after an initial diagnosis did not align with their experiences: *“[I] went to a walk-in clinic… they thought it was a bruise and they wanted to check me for a [deep vein thrombosis]. I wasn’t really satisfied with that, so I went to my dermatologist"(14).* Another participant described visiting multiple providers before eventually seeing a provider who recognized calciphylaxis: *“I [had] an appointment with my rheumatologist… I went to the primary doctor, he [thought] it may be shingles… [My doctor] said,"Go to the dermatologist and have them biopsy it” (15.)* This participant never got their biopsy results and was diagnosed with calciphylaxis about a month later when their nephrologist recognized the lesions. Another participant began chemotherapy because of misdiagnosis: *“In February, she began to treat me with a medicine for cancer”* (4). Participants had to be proactive and persistent in their pursuit of a diagnosis, which resulted in delayed and improper treatments because it was unclear who in the medical community could help them.

Pain and open lesions were the driving factors for hospital admission before diagnosis. One participant had multiple hospitalizations for pain before being diagnosed with calciphylaxis. The doctors had “*no idea what’s going on. I was in the hospital two times in… January for various issues trying to figure out what’s wrong with me. Then February, the same thing” (3).* Often, providers did not grasp the extent of participants’ pain and questioned whether it was real at all: *“I got the real impression that [the doctors] didn’t understand my pain. They really questioned whether I was in pain”* (5). In other instances, participants’ frequent visits to the ED for pain were attributed to drug-seeking behavior: “*I had a lot of issues with people understanding what was going on with me. I wasn’t a drug seeker. I wasn’t trying to get medication, but I had terrible pain… I had to explain it to everybody that I encountered—even the doctors*” (11). Participants’ early experiences with care were characterized by misdiagnosis, incorrect treatments, and realizations about the lack of awareness and knowledge about calciphylaxis. These experiences were reinforced by their interactions with providers.

### Case management and calciphylaxis literacy

A common experience among participants was the realization that medical providers lacked calciphylaxis education and knowledge. Part of the participants’ understanding of calciphylaxis was medical providers’ lack of knowledge about it, *“I don’t think they [ER doctors] knew exactly what to do… I’ve learned… [calciphylaxis] is not a condition a lot of people know about”* (7). Even among nephrologists, calciphylaxis knowledge is limited. One participant described how this led to them feeling unable to rely on their providers: *“[The dermatologist’s] position was,"We don’t know a lot about calciphylaxis, go to your nephrologist."[The] nephrologist said,"We don’t see this a lot, so we don’t know a lot."They relied on their ignorance”* (15). Participants often did not understand the severity of their condition because providers could not explain calciphylaxis. In one instance, a participant grossly misunderstood their diagnosis because, *“[the doctor] didn’t explain anything and I thought,"Okay. Calciphylaxis, it’s a condition I have. [The doctor will] do something to cure it"* (13). The breadth of calciphylaxis illiteracy among providers led to miseducated patients.

Despite lack of general knowledge about calciphylaxis, referrals to other physicians were relatively limited. This resulted in participants having to self-educate and self-advocate: *“I had to [study calciphylaxis] because nobody else around me knew anything. No disrespect to the doctors, but I had to. I was left to my own devices” (15).* This gap in understanding also resulted in uncertainty about which providers to seek care from. Disparate calciphylaxis knowledge negatively impacted the patient experience.

When providers discussed calciphylaxis, they emphasized its high mortality rate. This emphasis on mortality was frustrating and demoralizing for participants; *“When someone is diagnosed with calciphylaxis, they don’t know what to expect. When they Google it, it is like… reading their death warrant…* (15). When providers could not tell a patient about what a diagnosis of calciphylaxis entailed, participants were left to discover calciphylaxis on their own.

Participants in this study became their own advocates in the clinic, suggesting treatments and doing their own wound care. One participant described their efforts to find a provider as an interview process: “*I interviewed other doctors… I asked them how many calciphylaxis patients have they treated or have they dealt with”* (3). Another participant shared *“I think I’m actually the one that healed myself… they wouldn’t give me saline… [so] I made my own*” (10). Providers could not explain expectations regarding treatment, pain, and impact on daily life, leaving participants to feel isolated and disheartened.

### Challenges posed by provider’s calciphylaxis illiteracy

The lack of calciphylaxis knowledge among providers impacted participants’ clinical care. For one participant, “*I got rid of the first doctor I had… because you could tell he just didn’t care… My second nephrologist told me, ‘Oh, we only do [sodium thiosulfate for] … 12 or 16 weeks… I got rid of [that doctor] ‘cause he wasn’t gonna treat me anymore with [sodium thiosulfate]”* (3). In one case, a provider’s inexperience and lack of referral led one participant to be put on dialysis. Their doctor believed that calciphylaxis was terminal but *“he did not want to be the one to tell me that it was terminal… He did not want to tell me that he wasn’t capable of treating it and he didn’t know that much about it”* (8). Later, this participant was told by another doctor that surgical debridement may help them. This participant shared that they wished they “*had never been put on dialysis. I wish there [were] more options back then*” (8). Conflicting information has severe, lasting impacts on participants’ lives.

Because providers could not answer participants’ questions about calciphylaxis, participants learned about calciphylaxis on their own. One participant *“started with Epic. I started looking for definitions… I did Google straight up”* (6). After being diagnosed, another participant’s provider *“didn’t get into detail with me at all. I had to do the research on my own and find out how rare it was, that there was no cure for it” (13).* Another participant had a recommendation for calciphylaxis patients: “*Most of all, do your own research on this disease. That’s an absolute must*” (3) because providers do not know very much.

The lack of knowledge about calciphylaxis significantly impacted pain management. Participants reported that most of their providers were reluctant or unable to prescribe pain medication and did not refer patients to a provider who could prescribe medication. One participant described a typical experience as “*you go to the hospital and people there don’t understand and don’t take you seriously, it’s frustrating…. I needed pain medicine, and they would look at me like I was some druggie*” (11). Even when pain medications were used, including opioid and narcotic-based medication, participants reported that they still had debilitating pain: “*It’s constant pain. It never goes away. It’s not like I really get relief from it. It’s always there, even with all the pain medication*” (13). Providers’ lack of knowledge about calciphylaxis combined with the dismissal of participants’ pain was experienced as bias, stereotyping, and stigma related to assumed drug-seeking, both in outpatient and emergency room settings.

As a result of medication bias, participants adopted a variety of tactics to self-advocate for pain control. One participant was prescribed medication after they got their wound care doctor to discuss the issue with their primary doctor. This participant explained, *“I have a new [primary doctor] who doesn’t want to order anything until you explain to him… I think the wound doctor spoke to him”* (11). Sometimes providers seemed to not trust their patients. One participant *“wrote a letter to [my doctor’s] attending… saying I thought I was being treated like a drug addict and this had to change*” (12). This discrimination when requesting pain medication led participants to feel alienated by the medical community and contributed to the development of the self-advocate role embraced by our participants.

### Participants responses to calciphylaxis

Calciphylaxis pain was an omnipresent aspect of participants’ experiences. As one participant explained, the pain *“didn’t let me live. It’s all the time… Sometimes, the medicine diminish… but don’t disappear…”* (4). Calciphylaxis changed participants’ engagement in social activities and employment. One participant expressed a common occurrence among study participants in that they *“[had] to stop working because I can’t walk up steps… It’s like I can’t go back to a normal life*” (9). Participants’ lives became about surviving the pain and making it from one day to the next.

Calciphylaxis austerely impacted participants’ lives. In response to the pain*, “I stopped even reading. I stopped eating, and I couldn’t do anything. I had so much pain… I didn’t have a normal life”* (4). Reliance on family was an experience that many participants shared. For some, this dependence came with complicated emotions: *“It’s hard to not see years coming off of my parents’ lives… I know I have such a great support system… [I feel like] I’m burdening them.”* (14). Another participant described that “*being in a wheelchair [due to calciphylaxis] was very frustrating. You have to depend on somebody*” (2). Although the emotions surrounding support systems could be complicated, participants who had support had profound gratitude for their support. One participant summarized what many others reported: *“I needed that support system. I don’t think I would’ve recovered… without that”* (1). When experiencing *“[calciphylaxis] you put living on hold. You are surviving. Day to day. Minute to minute. Hour to hour”* (15), a sentiment echoed by many participants. The challenges posed by calciphylaxis, including the pain and uncertainty, make social support a key factor in recovery and coping.

Experiencing calciphylaxis impacted the physician–patient relationship. Due to the lack of calciphylaxis knowledge among providers, “*Anybody I come in contact with that just doesn’t understand [calciphylaxis], I explain it to them in—some people you look at them and it goes in one ear and out the other”* (11). Some providers did not respond to calciphylaxis as the participant had hoped. One participant shared that *“I had a lot of respect for [my PCP], but with all this going on, I’m losing my respect fast”* (13). The general lack of knowledge about calciphylaxis and the perceived ambivalence towards calciphylaxis patients led some participants to lose trust in their medical providers.

## Discussion

This qualitative study of 15 patients with calciphylaxis provides comprehensive insights into the lived experiences of patients who suffer from a rare, severe illness. In this study, we identify and describe three dimensions of the calciphylaxis experience; knowledge, or lack thereof, of calciphylaxis and its treatment, disruptive socio-emotional impacts of having a painful, poorly understood medical condition, and subsequent impacts on the provider-patient relationship.

While we readily acknowledge that systemic factors impact a provider’s ability to treat calciphylaxis—including the rarity of calciphylaxis, regulatory limitations regarding opioid prescription, and demands on physician knowledge, the breadth of knowledge required of medical providers—our findings about patients’ calciphylaxis experience suggest several clinical recommendations that could improve patients’ care around other acutely severe illnesses that are less commonly observed. For example, learned from these participants’ experiences could be used to help not only calciphylaxis patients lead more fulfilling lives but also provide insights into the experiences of care around conditions such as acute leukemias or rapidly progressive glomerulonephritis that need prompt treatment for successful outcomes^12,13^.

The dimension of knowledge is central to the lived experience of calciphylaxis. There is insufficient research and knowledge available about calciphylaxis^1,14^. The lack of calciphylaxis knowledge has significant implications for pain management. From the participants’ perspective, providers’ lack of education about calciphylaxis results in providers not understanding calciphylaxis pain and showing limited empathy towards patients. Although medical understanding of calciphylaxis is limited, the severity of calciphylaxis pain and insensitivity to medication is well-established. However, providers were reluctant to prescribe medications that mitigate pain. From the participants’ perspective, this reluctance is motivated by assumptions about opioid addiction or drug-seeking behavior. Participants experienced discrimination when self-advocating for better pain management, especially in emergency departments. Assumptions leading to stigma about the need for pain management should not outweigh the evidence that calciphylaxis is incredibly painful.

It is well known by providers that the personal burden, both medically and emotionally, of calciphylaxis is extensive. A brief qualitative study by Singh et. al published in 2022 describes the primary burden of calciphylaxis as the pain patients experience^15^. Our study corroborates this finding, including the lack of referrals to pain management specialists, while expanding on how pain impacts patients’ daily life. Beginning with seeking avenues to manage pain, calciphylaxis soon occupies most every aspect of a patient’s existence, from seeking to alleviate pain to hospitalization for pain to causing social withdrawal and isolation from friends and family. The extent to which calciphylaxis invades every aspect of a patient’s life really cannot be overstated.

The socio-emotional impacts of having a rare, severe condition that is insufficiently understood by the medical community constitute another domain of experience. Calciphylaxis takes a significant toll on the body and one’s mental and emotional state due to the condition’s pain, morbidity, and uncertainty. The lack of knowledge about calciphylaxis among providers and omnipresent pain appears to be the root cause of much of the uncertainty and frustration experienced by participants. Resoundingly, participants expressed frustration that there was no straightforward way for providers to help them. There was a sense among some participants that providers did not take them seriously and discounted them as ‘lost causes.’ When providers do not appear to take patients’ concerns seriously, cannot adequately manage their pain, and cannot educate patients about their diagnosis, it is no surprise that participants can become depressed, anxious, fearful about their future, and question the utility of their relationship with their medical provider.

Alterations in the patient-physician relationship characterize the third domain of experience and calciphylaxis burden. Trust, knowledge, regard, and loyalty have been identified as important aspects of a positive physician–patient relationship^16^. In the guidance-cooperation model of patient-provider relationships, which is the typical model of the American healthcare system, physicians have the medical knowledge, and therefore the power, in the relationship^17^.This study demonstrates that physicians often do not have calciphylaxis knowledge, resulting in reduced trust in providers. To fill this knowledge vacuum, patients take it upon themselves to learn about calciphylaxis, often becoming more knowledgeable about their condition than their providers. Thanks to their newfound knowledge, patients are empowered to be self-advocates. The shift in knowledge level has the potential to significantly alter the patient-physician relationship.

Given the unique circumstances of calciphylaxis, it is important to acknowledge the potential role of cognitive biases. Due to the rarity of calciphylaxis, providers may experience variable forms of bias, such as anchoring bias, in which their initial diagnosis is incorrect, and symptoms are attributed to previously known factors, such as diabetes. Alternatively, physicians may experience confirmation bias, which involves ignoring contradictory evidence while only giving merit to supportive evidence, when reaching a diagnosis. Confirmation bias may play a role in the difficulty of some participants in obtaining adequate pain management. An undiagnosed calciphylaxis patient seeking pain relief asking repeatedly for pain control to no avail may appear to exhibit drug-seeking behaviors to a provider who is unfamiliar with calciphylaxis. To combat bias, Doherty et al. recommend utilizing the dual-process theory during clinical decision making^18^. This cognitive model emphasizes the differences between intuitive thinking and analytical reasoning. In the case of calciphylaxis, relying more on analytical reasoning, which would push physicians to be overly thorough in their evaluations, may yield more rapid diagnosis of calciphylaxis and, in turn, better treatment. Additionally, analytical thinking may push physicians to truly deep dive into what is causing their patients such intense pain, which would ultimately result in improved pain management. Furthermore, empathy has been shown to improve not just the patient experience but also the provider experience^19^. Practicing empathy may be another tool to reduce biases experienced during calciphylaxis^20^.

The results of this study inform several directions for future calciphylaxis and rare disease research and have implications for clinical practice. Regarding future research, this study, as the first of its kind, has established a basic understanding of the calciphylaxis patient experience. This experience hinges on the state of calciphylaxis education among physicians, communication with physicians, emotional responses to the condition, and extensive disease burden, specifically struggles with pain management. Avenues for future research include exploring the role of caregivers, physician perspective, and the patient-physician relationship in rare, severe diseases. Given the current hostile and biased environment surrounding opioid prescriptions^21–23^, future research about pain management accession in calciphylaxis and other painful conditions is warranted.

This study yields four key clinical implications. Firstly, given the state of calciphylaxis knowledge among physicians, the utilization of a shared decision-making model of health care, which has been shown to positively impact patient outcomes, should be explored for calciphylaxis care^24,25^. A shared decision-making model of healthcare may help patients feel respected and heard, two issues calciphylaxis patients sometimes experience. Skilled physician–patient communication, which is essential to the shared decision-making model^26,27^, is integral to positive physician–patient relationships^28^. This study demonstrates that, even when the state of knowledge is limited, it is better to share that state of knowledge with patients, however limited or incomplete it may be, than to not address it at all. Emphasizing education around difficult conversations during medical training and a physician’s career could have positive implications for patients’ disease experiences^29^. Thirdly, this study indicates a need for comprehensive, easily accessible calciphylaxis educational tools for both patients and providers. This tool could take various forms, such as videos by patients describing the disease experience. Developing a document describing the minimum knowledge necessary for addressing potential calciphylaxis could be shared and distributed to nephrology and dermatology clinics or be made available on commonly accessed medical resources, such as UpToDate, for physicians to readily access. Finally, this study contributes to the understanding that calciphylaxis is a complex condition. To address this experience, we recommend that mental health specialists and pain management specialists be included on a calciphylaxis care team.

## Limitations

This study has several limitations. All participants in this study were healthy enough to participate (i.e. not hospitalized or in hospice) and so the results of this study do not directly address the hospitalized or hospice calciphylaxis experience. The lack of additional variables, such as socioeconomic status and comorbidities, is also a limitation.

Although this study was run from and recruited through a large, academic medical center in a large city in the Northeast United States, participants were from across the Northeast, Midwest, and Southern US. Due to the medical center’s specialty in calciphylaxis, patients from across the US seek treatment there. There may be disparities in care and calciphylaxis expertise in different cities, and even hospitals within one city, which study does not account for. Avenues for further research to address this include comparing care in rural versus urban settings and even across the world. The results of this study are not the only calciphylaxis experience but rather encompass just one perspective, likely one that many patients share, of the calciphylaxis experience.

## Conclusion

Calciphylaxis is a life-altering condition characterized by severe pain and uncertainty on multiple fronts. This study demonstrates that clinical management of rare, severe illnesses, such as calciphylaxis, requires clear, honest provider-patient communication, sufficient provider and patient education, adequate symptom management, and empathy.

## Data Availability

Data is provided within the manuscript. The interview guide and de-identified interview transcripts are available upon request to the corresponding author.
